# Ag43-mediated display of a thermostable *β*-glucosidase in *Escherichia coli* and its use for simultaneous saccharification and fermentation at high temperatures

**DOI:** 10.1186/s12934-014-0106-3

**Published:** 2014-08-01

**Authors:** Iván Muñoz-Gutiérrez, Cessna Moss-Acosta, Berenice Trujillo-Martinez, Guillermo Gosset, Alfredo Martinez

**Affiliations:** 1 Departamento de Ingeniería Celular y Biocatálisis, Instituto de Biotecnología, Universidad Nacional Autónoma de México, A. P. 510-3, Cuernavaca, 62250, Mor, México; 2 Present address: Department of Molecular Microbiology and Biotechnology, George S. Wise Faculty of Life Sciences, Tel Aviv University, Tel Aviv, Israel

**Keywords:** Ethanologenic Escherichia coli, Simultaneous saccharification and fermentation, Cellulose, Autotransporter Ag43, Thermobifida fusca β-glucosidase, Ethanol

## Abstract

**Background:**

The autotransporter (AT) system can potentially be used in the secretion of saccharolytic enzymes for the production of lignocellulosic biofuels and chemicals using *Escherichia coli*. Although ATs share similar structural characteristics, their capacity for secreting heterologous proteins widely varies. Additionally, the saccharolytic enzyme selected to be secreted should match the cell growth or cell fermentation conditions of *E. coli*.

**Results:**

In the search for an AT that suits the physiological performance of the homo-ethanologenic *E. coli* strain MS04, an expression plasmid based on the AT antigen 43 (Ag43) from *E. coli* was developed. The *β*-glucosidase BglC from the thermophile bacterium *Thermobifida fusca* was displayed on the outer membrane of the *E. coli* strain MS04 using the Ag43 system (MS04/pAg43BglC). This strain was used to hydrolyze and ferment 40 g/L of cellobiose in mineral media to produce 16.65 g/L of ethanol in 48 h at a yield of 81% of the theoretical maximum. Knowing that BglC shows its highest activity at 50°C and retains more than 70% of its activity at pH 6, therefore *E. coli* MS04/pAg43BglC was used to ferment crystalline cellulose (Avicel) in a simultaneous saccharification and fermentation (SSF) process using a commercial cocktail of cellulases (endo and exo) at pH 6 and at a relatively high temperature for *E. coli* (45°C). As much as 22 g/L of ethanol was produced in 48 h.

**Conclusions:**

The Ag43-BglC system can be used in *E. coli* strains without commercial *β*-glucosidases, reducing the quantities of commercial enzymes needed for the SSF process. Furthermore, the present work shows that *E. coli* cells are able to ferment sugars at 45°C during the SSF process using 40 g/L of Avicel, reducing the gap between the working conditions of the commercial saccharolytic enzymes and ethanologenic *E. coli*.

## Background

The approaching exhaustion of abundant, inexpensive oil and the pollution produced by burning fossil fuels are both factors that are driving research to explore environmentally friendly renewable fuels [1]. Different sugar sources can be transformed by fermentation processes into liquid biofuels, such as bioethanol, that can be used, after distillation and dehydration, in current internal combustion engines [1]. Lignocellulose, the most abundant biopolymer on earth, represents a source of fermentable sugars that does not compete with the production of foodstuffs. The transformation of lignocellulose into ethanol fuel consists of several steps: 1) pretreatment of the biomass to reduce the crystallinity of the cellulose, to eliminate a fraction of the lignin and to hydrolyze part of the sugars contained in the hemicellulosic fraction, 2) enzymatic hydrolysis of the pretreated material to depolymerize most of the cellulosic fraction, 3) fermentation of the released mixture of sugars, and 4) ethanol purification [2]. To simplify the production process and reduce costs, enzymatic hydrolysis and fermentation can be performed in a single reactor (simultaneous saccharification and fermentation (SSF)). The SSF process reduces the number of reactors needed and avoids the end product inhibition of cellulases [3].

The hydrolysis of lignocellulose produces a wide variety of fermentable sugars, such as hexoses, pentoses and uronic acids, and a capable microorganism is required to ferment all of these sugars. *Escherichia coli* has this ability, and has been metabolically engineered to create homo-ethanologenic version of this bacterial strain [4]-[7]. Although it is possible to perform the SSF process with pretreated lignocellulose using *E. coli*, the major drawback is that commercial enzymes and *E. coli* are incompatible in terms of their working conditions. While commercial enzymatic cocktails work at pH 5 and 50°C [8], *E. coli* grows at pH 7 and 37°C. As a consequence, a balance in the pH and temperature must be found. In a recent report, our research group showed the ability of the ethanologenic *E. coli* strain MS04 [5] to ferment pretreated corn stover in a SSF process that occurred at 37°C and pH 6 [9], reducing the gap between the saccharification conditions of commercial saccharolytic enzymes and the fermentation conditions of *E. coli*. Although ethanologenic *E. coli* MS04 showed good compatibility and performance with the commercial cocktail of cellulases, it is necessary to reduce the amount of externally added enzymes during the SSF process [10]. To achieve this reduction, *E. coli* must be engineered to secrete saccharolytic enzymes. In this regard, the cell surface display of enzymes involved in the depolymerization of low-cost polysaccharides is attracting much attention [10].

One option for cell surface display in *E. coli* is the type V secretion system, also known as the autotransporter (AT) system. ATs are multidomain proteins consisting of a signal peptide (SP), a passenger protein and a translocation unit (TU). The TU consists of a linker region and a β-barrel [11],[12]. The linker region anchors the passenger protein to the cell surface. However, some ATs process the linker region after the passenger protein is translocated across the outer membrane, releasing the passenger protein into the milieu; it can also remain strongly associated with the TU [11],[12]. To display a heterologous protein with the AT system, the DNA sequence of the native passenger protein is replaced by the heterologous sequence [11]. The relative simplicity of the AT system makes it potentially useful for the secretion of saccharolytic enzymes in *E. coli* strains engineered for the production of biocommodities [10]. Nevertheless, there are only a few reports that show that the secretion of saccharolytic enzymes is possible using the AT system with this bacterium [13],[14]. Although the ATs share similar structural characteristics, their capacity for secreting heterologous proteins varies widely depending on the genetic background of the *E. coli* strain that is used [15]-[18]. Additionally, the performance of the cell surface display can be affected by the composition of the media, the presence of proteases, the temperature and the cultivation technique used, among other factors [15]. As a consequence, it is important to find an AT that can be adapted to specific conditions. Gram-negative bacteria have seven specialized protein secretion systems (types I to VI and the chaperone–usher pathway) that allow secreted proteins to travel through the inner and outer membrane [12]. Autotransporters are a superfamily of proteins that use the type V secretion pathway for the delivery of proteins to the surface of Gram-negative bacteria [12]. A bioinformatics study performed by Wells *et al*. in 2010 [19], with twenty-eight *Escherichia coli* genomes, revealed that *E. coli* has 215 autotransporter-encoding sequences.

In this work, in the search for an AT that suits the genetic background and physiological performance of the ethanologenic *E. coli* MS04 strain, we constructed an expression plasmid based on the AT antigen 43 (Ag43) from *E. coli*[20]. To facilitate the cloning of any gene encoding a saccharolytic enzyme, we introduced a multiple cloning site between the SP and the TU of Ag43. The *β*-glucosidase BglC from the thermophile bacterium *Thermobifida fusca*[21], an enzyme that possesses a high robustness when secreted in *E. coli*[13],[22]-[24], was displayed using the Ag43 system. Taking advantage of the fact that BglC shows its highest activity at 50°C and retains more than 70% of its activity at pH 6 [21], *E. coli* MS04 expressing the Ag43-BglC system was used to ferment crystalline cellulose (Avicel) in an SSF process with a commercial cocktail of cellulases at pH 6 and at a relative high temperature for *E. coli* (45°C). The present work shows that the Ag43-BglC system can be used in ethanologenic *E. coli* strains to avoid the use of commercial *β*-glucosidases, reducing the amount of commercial enzymes used during SSF processes. Further, it shows that ethanologenic *E. coli* cells can be used to ferment sugar mixtures at 45°C.

## Results

### Construction of the Ag43 display system

**Figure 1:**
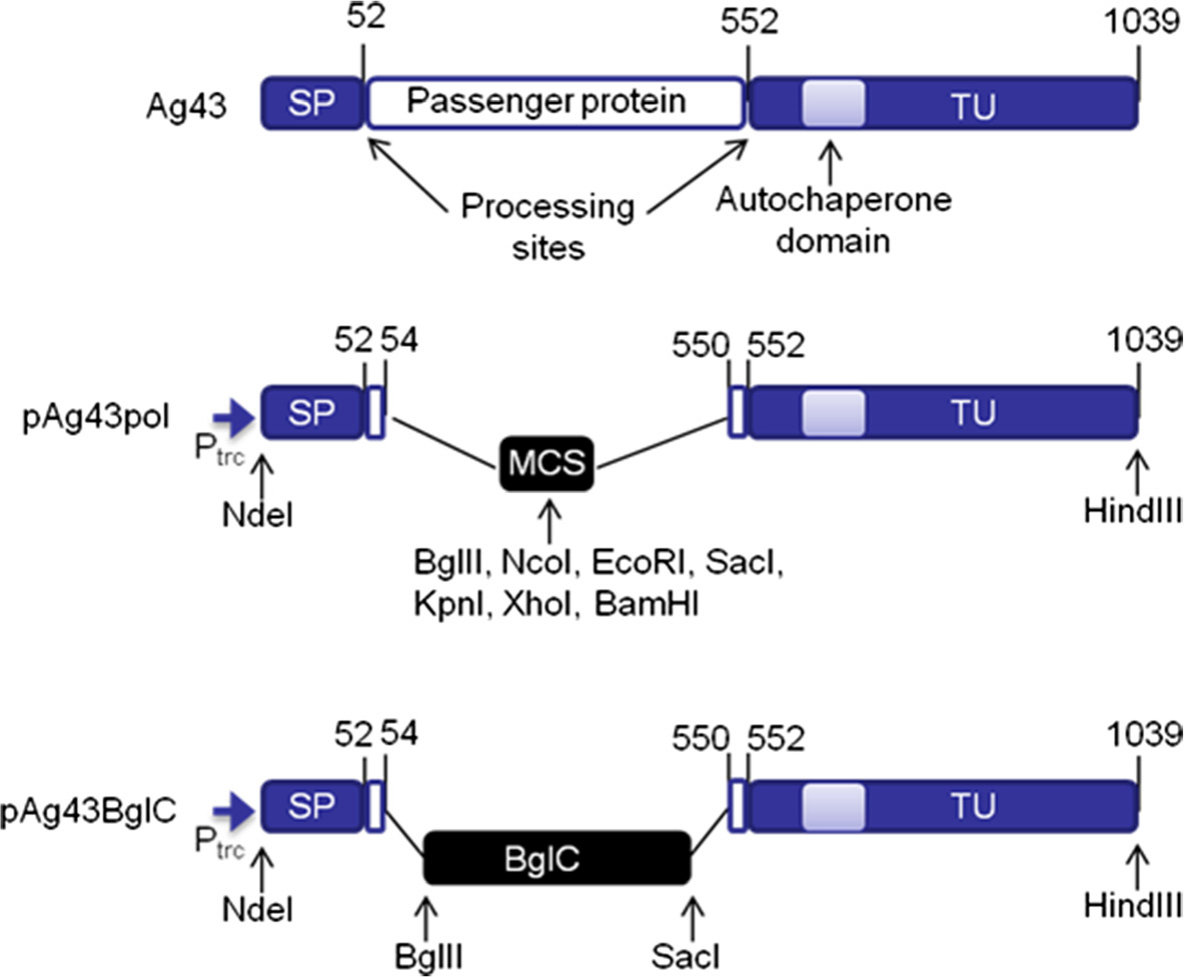
**The arrangement of the protein domains of Ag43 and an overview of the Ag43-based secretion systems developed in this study (drawing is not to scale).** The numbers designate the amino acids at the domain boundary. The restriction sites used for plasmid construction are indicated. SP, signal peptide; TU, translocation unit; P_trc_, promoter trc; MCS, multiple cloning site; BglC, *T. fusca β*-glucosidase.

Figure 1 shows the domain organization of Ag43 and depicts the structure of the developed plasmids pAg43pol and pAg43BglC. The Ag43 domains and the relevant restriction sites used during plasmid construction are shown. The pTrc99A2 plasmid (Table 1), which contains the IPTG-inducible *trc* promoter, was used as the backbone of the Ag43-based secretion system. During the construction of the pAg43pol plasmid, the RfluBNE and DfluKXB primers inserted the unique restriction enzyme sequences *Bgl*II, *Nco*I, *EcoR*I, *Kpn*I, *Xho*I and *BamH*I, creating multiple cloning sites between the SP and the TU. The pAg43pol plasmid contains the native Ag43 SP and the sequence that encodes amino acids 550 through 1039 of Ag43, including the TU and the autochaperone domain [20]. Whereas some ATs designed for the display of heterologous proteins replace the native SP, such as the widely used AIDA-I system developed by Maurer *et al*. [16], the pAg43pol plasmid contains the native Ag43 SP. Some ATs, such as Ag43, have unusually long SPs that likely slow the translocation of the ATs across the inner membrane, preventing the accumulation of misfolded proteins in the periplasmic space [12]. To avoid cleavage at amino acid 552 and keep the heterologous passenger protein attached to the cell surface, the aspartyl protease active site present in the Ag43 passenger protein [25] was not included. To display BglC on the surface of *E. coli* MS04, the *bglC* gene was cloned into pAg43pol to obtain pAg43BglC as described in the Methods section.

**Table 1:** ***E. coli*** **strains, plasmids and primers used in this study**

**Strain**	**Relevant genotype**	**Source**
*E. coli* DH5α	RecA	Promega
*E. coli* MG1655	Wild type	Laboratory stock
*E. coli* MS04	MG1655 ∆*pfl*B, ∆*adh*E, ∆*frd*A, ∆*xyl*FGH, *gatC S184L, ∆midarpA, ∆reg 27.3 kb,* ∆*ldh*A, P*pfl*B::*pdc*_*Zm*_-*adh*B_*Zm*_.	[5],[28]
*E. coli* BL21 (DE3)	F^−^*ompT hsdS*_*B*_ (*r*_*B*_^*−*^*m*_*B*_^*−*^) *gal dcm* (DE3)	Novagen
**Plasmid**	**Description**	**Source**
pTrc99A2	pTrc99A derivative modified for *Nde*I cloning instead of *Nco*I. Designed for IPTG-inducible expression of proteins under the hybrid *trp/lac* promoter. Amp^r^.	[13]
pAIDABglC	pTrc99A derivative designed for cell surface display of BglC using the autotransporter AIDA-I. Amp^r^.	[13]
pET-22b(+)	Designed for IPTG-inducible expression of proteins under the T7 promoter. Amp^r^.	Novagen
pETflu	pET-22b(+) derivative that has cloned the *flu* gene.	This study
pAg43pol	pTrc99A derivative designed for cloning heterologous sequences for protein secretion using the autotransporter Ag43. Amp^r^.	This study
pAg43BglC	pAg43pol derivative designed for cell surface display of BglC using the autotransporter Ag43. Amp^r^.	This study
pNS6	pET-26b(+) derivative employed for intracellular heterologous production of BglC in *E. coli*.	[21]
^**a**^**Primer**	**Sequence 5′ → 3′**	**Source**
DfluNde	CTAAGGAAAAG*CATATG*AAACGACATCTG	This study
RfluHind	CAGAGAGGCG*AAGCTT*CTGTCAGAAGGTC	This study
Pet38F (26)	CGATCTCGATCCCGCGAAATTAATAC	This study
RfluBNE	CGGT*GAATTC*CG*CCATGG*C*AGATCT*GTCAGCAGCCAGCACCGGGAG	This study
DfluKXB	GACC*GGTACCCTCGAG*CTGAAC*GGATCC*ATTGACCCCACG	This study

^a^The restriction sites employed during plasmid construction are *underlined*.

### Whole-cell protease treatment analysis

**Figure 2:**
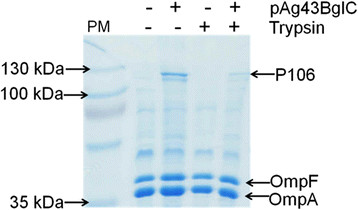
**SDS-PAGE showing the protease accessibility of BglC attached to the surface of the outer membrane of*E. coli*MS04.** PM refers to the protein marker; P106 is the protein product of pAg43BglC without the SP and represents the sum of Ag43 TU and BglC. The plasmid pTrc99A2 was employed as a negative control. The (−) symbol in the pAg43BglC line means that the proteins were from cultures grown with MS04/pTrc99A2. The (−) symbol in the trypsin line means OMPs of cells not treated with trypsin. Note that OmpC, which migrates together with OmpF, is not found in the figure because during the development of strain MS04, an adaptive evolution step to improve the consumption and growth on xylose was developed, and a region of the genome that contains the *ompC* gene was lost [5],[28].

Proteins that are attached and exposed externally on the cell surface of *E. coli* can be removed from whole cells using proteases. After proteolytic treatment, outer membrane proteins (OMPs) can be purified and analyzed using SDS-PAGE, at which point little or no cell surface proteins will be observed. To show that BglC is exposed externally on the cell surface of MS04, whole cells expressing the Ag43-BglC system were treated with trypsin as reported previously [13]. Cells carrying pTrc99A2 were used as a negative control. After their growth, the cells were harvested and “shaved” with trypsin, and the OMPs were subjected to purification. The calculated molecular weight of the chimeric protein Ag43-BglC without the SP is 106 kDa (thus, it was named P106). Figure 2 shows a polyacrylamide gel with the OMPs purified from both trypsin-treated and untreated cells. It can be observed that, in contrast with the negative control, the cells carrying the pAg43BglC plasmid that were not trypsinized possessed the protein P106. When the cells were treated with trypsin, the protein P106 content is drastically reduced, showing that the Ag43 system attaches BglC to the surface of *E. coli* MS04. In previous work involving the AIDA-BglC system [13], we performed both SDS-PAGE and western blot analyses of the outer membrane proteins and, as with the Ag43-BglC system, the SDS-PAGE clearly showed the identity of the fusion protein.

### Fermentation of glucose, cellobiose and cellulose

**Figure 3:**
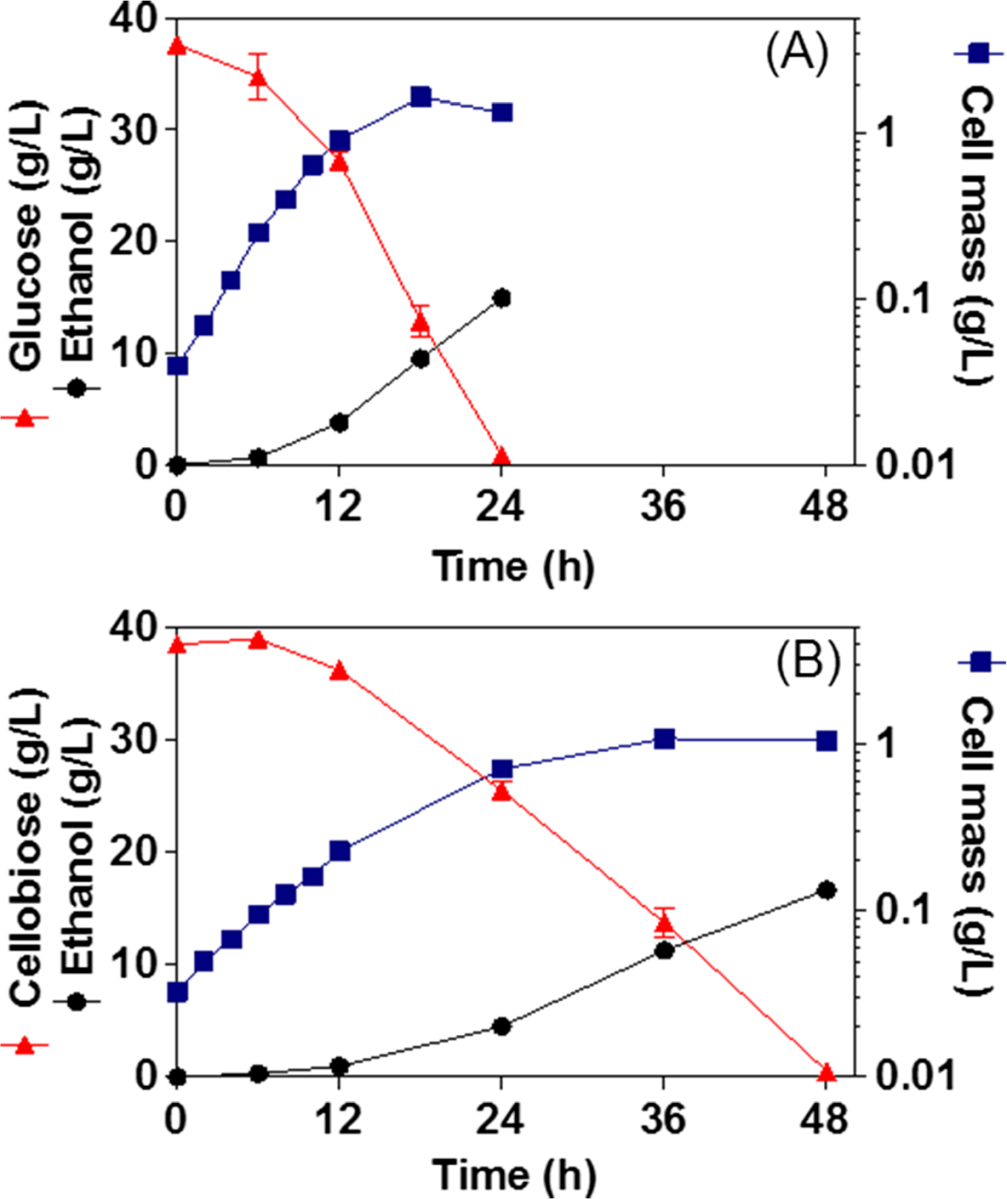
**Fermentation kinetics of glucose (A) and cellobiose (B) with MS04/pAg43BglC.** Cultures were performed at 37°C and pH 7 (n = 4).

*E. coli* MS04 expressing the Ag43-BglC system was tested for the hydrolysis and fermentation of 40 g/L glucose or 40 g/L cellobiose in mineral media. Figure 3 shows cell growth, glucose or cellobiose consumption and ethanol production during the fermentation processes. *E. coli* MS04/pAg43BglC had a specific growth rate (*μ*) of 0.276 h^−1^ (±0.005) in glucose and 0.153 h^−1^ (±0.004) in cellobiose; it consumed the glucose in 24 h and the cellobiose in 48 h and reached a titer of ethanol of 14.94 g/L (±0.64) with glucose and 16.65 g/L (±0.23) with cellobiose. The obtained ethanol yields were 80% (±3.2) and 81% (±2.6) of the theoretical maxima in glucose and cellobiose, respectively. During the fermentation of cellobiose, the *β*-glucosidase activities, either that present in the milieu or that was cell-associated, were measured at 37°C when the cells reached an OD_600_ of 1; values of 6.05 U/L (±0.88) and 59.6 U/g_DCW_ (±4) were obtained, respectively. The cell-associated activity represents 79% of the total activity, taking into account that 1 OD_600_ of cells is equal to 0.37 g_DCW_/L.

**Figure 4:**
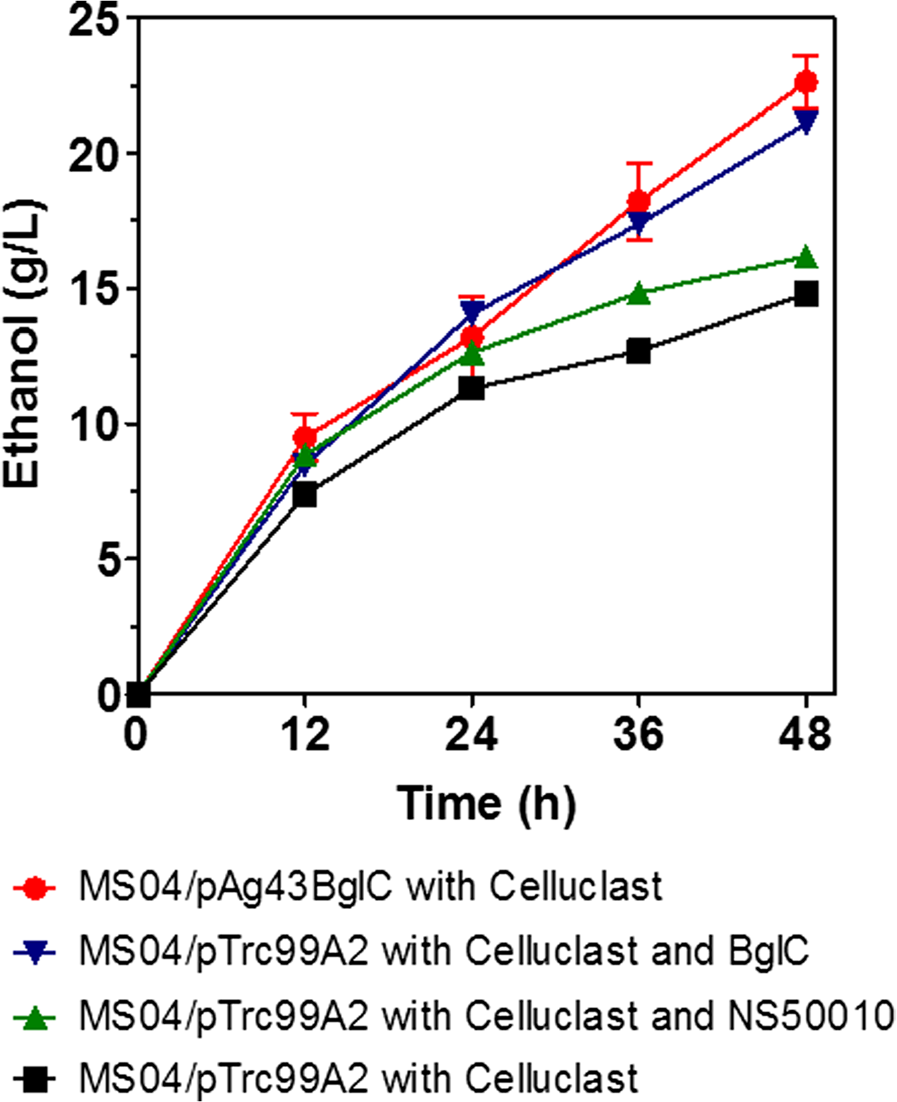
**Ethanol production during the SSF process of 40 g/L Avicel by MS04 carrying plasmid pAg43BglC or pTrc99A2.** These cultures were performed at 45°C and pH 6 (n = 3).

To show the compatibility of the displayed BglC with a commercial cocktail of cellulases and the capacity of *E. coli* MS04 to ferment at working conditions that can match these cellulases, *E. coli* MS04 expressing the Ag43-BglC system was tested in the SSF process with 40 g/L crystalline cellulose (Avicel) and the cellulase cocktail Celluclast (15 FPU/g Avicel of cellulases) at a temperature of 45°C and pH 6. Control experiments were performed using *E. coli* MS04/pTrc99A2 with Celluclast, *E. coli* MS04/pTrc99A2 with Celluclast and the *β*-glucosidase cocktail NS50010 (30 U/g Avicel of *β*-glucosidase) and *E. coli* MS04/pTrc99A2 with Celluclast and soluble *T. fusca* BglC (30 U/g Avicel of *β*-glucosidase produced from *E. coli* BL21 carrying plasmid pNS6, as mentioned in the Methods section). The ethanol production during the SSF of Avicel is shown in Figure 4. The highest titers of ethanol (approximately 22 g/L) were reached after 48 h of fermentation using MS04/pAg43BglC supplemented with Celluclast and the control MS04/pTrc99A2 supplemented with Celluclast and *T. fusca* BglC. The lowest titers of ethanol (approximately 15 g/L) were obtained using MS04/pTrc99A2 supplemented with Celluclast and MS04/pTrc99A2 supplemented with Celluclast and NS50010. It is interesting that the commercial cocktail of *β*-glucosidases (NS50010) did not improve the SSF process at the conditions tested. During the preparation of the inoculum for the SSF, *E. coli* MS04/pAg43BglC had an activity of 10.4 U/DO (measured at 45°C and pH 7).

## Discussion

The AT system has been extensively used for the heterologous display of proteins in *E. coli*[11]; however, the secreting capacities of heterologous proteins in ATs vary widely depending on the genetic background of the strain used [15]-[18] and the culture conditions [15]. Among ATs, Ag43 is a promising secretion system because it is able to translocate folded passenger proteins. Additionally, the outer membrane protease OmpT does not have a negative effect on the display of the passenger protein [17],[18]. To determine the potential of the display system Ag43 in the genetic background of the ethanologenic *E. coli* MS04, we constructed the expression plasmid pAg43pol, which contains multiple cloning sites between the SP and the TU and allows for the easy cloning of genes encoding saccharolytic enzymes or other proteins (Figure 1).

The secretion of *β*-glucosidases by ethanologenic microorganisms during cellulosic biofuel production allows the addition of commercial *β*-glucosidases to be avoided, reducing production costs. It is critical to select saccharolytic enzymes to be secreted by *E. coli* that have working conditions that match the growth conditions of the *E. coli*. In addition, the selected *β*-glucosidases must be expressed with enough activity to allow the growth of the cells and the fermentation on cellobiose [13],[22]-[24]. The *β*-glucosidase selected for secretion was BglC from *T. fusca*[21]*,* an enzyme that presents a high performance compared with other *β*-glucosidases that have been secreted by *E. coli*[22],[24]. For example, of the six different *β*-glucosidases studied by Tanaka *et al.*[24] and the three different *β*-glucosidases studied by Desai *et al.*[22], BglC from *T. fusca* was either the sole *β*-glucosidase to allow the growth of *E. coli* using cellobiose as the sole carbon source or the one that promoted the highest growth. In the present work, we show that the AT Ag43 is able to display BglC on the surface of the ethanologenic *E. coli* strain MS04 (Figure 2) and that the enzyme that is attached is catalytically active (*E. coli* MS04 expressing the Ag43-BglC system fermented cellobiose as the sole carbon source in mineral media) (Figure 3B). Although BglC activity was observed in the supernatant, most of it was cell associated (79%); this result is important because in the SSF process whole-cell biocatalysts are produced and recovered for subsequent hydrolysis and fermentation. The Ag43 system is more efficient for the secretion of *T. fusca β*-glucosidase BglC compared with the AIDA-I system [13]. *E. coli* MS04 expressing the Ag43-BglC system had a 24% higher cell-associated *β*-glucosidase activity, fermenting 40 g/L cellobiose in 48 h instead of 72 h as with the AIDA-BglC system. When grown on cellobiose, *E. coli* MS04 expressing the Ag43-BglC system had a *μ* that was 21% higher than that obtained with MS04 expressing the AIDA-BglC system [13].

In the present report, we show the suitability of the Ag43-BglC secretion system for SSF processes employing *E. coli* and commercial cellulases. In Figure 4, it is clear that *E. coli* MS04 expressing the secretion system Ag43-BglC performed better during the SSF of crystalline cellulose compared to cells carrying the empty plasmid that were supplemented with commercial *β*-glucosidases. It is important to note that the SSF process was carried out at 45°C and pH 6, conditions that allow for both the cellulase cocktail and BglC to perform better. The activities of the *β*-glucosidases present in the commercial cocktails are severely affected by both pH and temperature [26],[27]. These results show that commercial *β*-glucosidases do not need to be used with the Ag43-BglC system during SSF processes with ethanologenic *E. coli*.

## Conclusions

The selection of the right secretion system and saccharolytic enzymes is crucial during the engineering of *E. coli*. In the present work, we show that the AT Ag43 can be used to successfully secrete the *β*-glucosidase BglC; better results were obtained than when the previously reported AT system AIDA-BglC was used. Additionally, most of the *β*-glucosidase activity obtained with the Ag43-BglC system was cell-associated, allowing the recovery of the whole-cell biocatalyst for subsequent SSF processes. Finally, the feasibility of performing SSF at a relatively high temperature for *E. coli* was shown using commercial cellulases and 40 g/L of Avicel. Cells from the strain MS04/pAg43BglC were able to produce 22 g/L of ethanol at 45°C and pH 6, reducing the gap between the working conditions of the commercial saccharolytic enzymes and ethanologenic *E. coli*.

## Methods

### Strains and plasmids

The strains, plasmids and primers used in this study are listed in Table 1. *E. coli* DH5α was used for plasmid propagation during plasmid construction. Analyses of *β*-glucosidase secretion and ethanol fermentation were performed using the *E. coli* strain MS04 [5],[28]. Standard procedures were employed for DNA isolation, PCR, restriction-enzyme digestion, cloning, ligation, transformations, and gel electrophoresis experiments [29]. Polymerase chain reaction (PCR) experiments were performed using the Elongase Enzyme Mix (Invitrogen, Carlsbad, CA, USA). Restriction enzymes and ligases were purchased from Fermentas (Thermo Fisher Scientific Inc., MA, USA). PCR and agarose-gel products were isolated and purified using the High Pure PCR Product Purification Kit (Roche, Mannheim, Germany). Each plasmid construction was verified by its restriction pattern in an agarose gel and via sequencing. Primer synthesis and DNA sequencing were performed by the Core Unit of Synthesis and Sequencing of DNA at the Biotechnology Institute-UNAM.

The *flu* gene sequence, which encodes Ag43, was PCR-amplified using the DfluNde and RfluHind primers (Table 1); genomic DNA from *E. coli* MG1655 was used as the template. The PCR product was digested using the restriction enzymes NdeI and SacI and cloned into pET-22b(+) to obtain the pETflu plasmid. The amplification of the sequences that codify for the SP and the TU of Ag43 were conducted using the pETflu plasmid as a template. The SP sequence was amplified using the pet38F(26) and RfluBNE primers, and the TU sequence was amplified using the DfluKXB and RfluHind primers. Following PCR amplification, the SP sequence was digested using NdeI and EcoRI and cloned into pTrc99A2. Next, the TU sequence was cloned into this derivative plasmid using KpnI and HindIII to obtain the pAg43pol plasmid (Figure 1). Finally, the *bglC* gene was removed from the pAIDABglC plasmid using BglII and SacI. This gene was then gel-purified and cloned into pAg43pol to obtain the pAg43BglC plasmid (Figure 1).

### Whole-cell protease treatment and OMP purification

Whole-cell trypsin treatment and outer membrane proteins (OMPs) purification of trypsin-treated and non-treated cell were performed as described elsewhere [13]. The OMPs were analyzed using 12% sodium dodecyl sulfate polyacrylamide gel electrophoresis (SDS-PAGE). The samples were mixed with 2X loading buffer, heated in boiling water for 5 min, and subjected to electrophoresis.

### Culture conditions

Batch fermentation of cellobiose (Sigma-Aldrich, MO, USA) with cells expressing the pAg43BglC system were conducted as described elsewhere [13] in mineral AM1 media [30] supplemented with 0.1 g/L of citrate (J.T. Baker, Avantor Performance Materials, NJ, USA), 2 g/L of sodium acetate (J.T. Baker), 100 mg/L of Carbenicillin and 10 μM isopropyl *β*-D-1-thiogalactopyranoside (IPTG, Sigma-Aldrich). Cultures were performed in 300-mL Fleakers mini-fermenters [31] containing 200 mL of media without aeration at a pH of 7, a temperature of 37°C and a speed of 150 rpm. Automatic additions of 2 N KOH preserved a constant pH. Prior to fermentation and the SSF process, the cells were adapted to grow in mini-fermenters as described previously [13]. The adapted cells were stored in cryotubes containing glycerol (40% final) to a final volume of 1.4 mL at −70°C.

To perform the SSF process with Avicel (PH-101, Sigma-Aldrich), a culture tube with 3 mL of Luria Broth (LB; 5 g/L yeast extract, 10 g/L Bacto peptone and 5 g/L NaCl), 100 μg/mL of Carbenicillin and 10 g/L of glucose was seeded with a cryotube containing cells of *E. coli* MS04/pAg43BglC or *E. coli* MS04/pTrc99A2 adapted into mini-fermenters The cells were grown at 37°C and 300 rpm for 4 h. Subsequently, the cells were inoculated in 1 L mineral media with 50 g/L of glucose and 100 mg/L of Carbenicillin at an initial OD_600_ of approximately 0.001 and grown in a 1-L fermenter (Applikon ADI 1010/ ADI 1025, Schiedam, The Netherlands) with an aeration rate of 0.1 vvm at a temperature of 37°C, a pH of 7.0 (adjusted by the automatic addition of 2 N KOH) and a speed of 400 rpm (using one Rushton Turbine with 6 blades). During the growth of *E. coli* MS04/pAg43BglC, the temperature was maintained at 30°C when the cells reached 0.5 OD_600_. In our previous work employing the autotransporter AIDA-I [13], we found that induction with 100 μM IPTG hindered cell growth in comparison to when 10 μM IPTG was used. For this reason, 10 μM IPTG was also used in mini-fermenter cultures with the autotransporter Ag43. However, during the experiments in 1-L fermenters, we found that when 20 μM IPTG was used for induction the cells produced enough β-glucosidase activity to continue the SSF process. Hence, MS04/pAg43BglC and MS04/pTrc99A2 cells grown in the 1-L fermenters were induced with 20 μM IPTG. Both strains were allowed to grow until 5.5 OD_600_ was reached, and then the cells were harvested by centrifugation at 4,000 × *g* for 10 min at room temperature (RT). Finally, SSF was carried out in 300-mL Fleakers containing 200 mL of 50 mM ammonium phosphate buffer with 40 g/L of Avicel, 0.2% Tween 80, 100 mg/L of Carbenicillin and 10 OD_600_ of cells. SSF was performed at 45°C, 150 rpm and pH 6 (adjusted by the automatic addition of 2 N KOH).

### Preparation of soluble BglC and enzyme assays

The production of soluble BglC was carried out with *E. coli* BL21 (DE3) carrying the plasmid pNS6 [21]. The cells were grown overnight in flasks containing 50 mL of LB with 30 μg/mL of Kanamycin at 37°C and 300 rpm. Subsequently, the 50 mL overnight culture was used to inoculate 950 mL of LB with 30 μg/mL of Kanamycin, and the cells were grown at 37°C and 300 rpm. When the cells reached an OD_600_ of 0.5, the incubation temperature was switched to 20°C and the cells were induced with 0.5 mM IPTG. Next, the cells were allowed to grow until an OD_600_ of 2 and harvested by centrifugation for 10 min at 4,000 × *g* and 4°C. The cells were washed twice with 20 mL of 50 mM phosphate buffer (pH 7), and the cell pellet was resuspended in 5 mL of 50 mM phosphate buffer (pH 7). Finally, the cells were lysed by sonication, and the *E. coli* extract was recovered by centrifugation for 30 min at 13,400 × *g* and 4°C. The extract was used as the source of soluble BglC during SSF control experiments with 30 U/g Avicel.

The commercial enzymes used during the SSF process were the cellulase cocktail Celluclast® 1.5 L (Sigma-Aldrich) and the *β*-glucosidase cocktail NS50010 (kindly provided by Novozymes, Brazil). Celluclast® 1.5 L and NS50010 were used during SSF at a concentration of 15 FPU/g Avicel and 30 U/g Avicel, respectively. The cellulase activity of Celluclast® 1.5 L was measured using a filter paper substrate, and the *β*-glucosidase activity of NS50010 was measured using a *p*-nitrophenyl-*β*-d-glucopyranoside (pNPG; Sigma) substrate. The activities of the commercial cocktails were calculated using the protocols described by Wood and Bhat [32].

The activity of the cell surface-attached *β*-glucosidase was assayed using a pNPG substrate. During the assays, 1 mL of cells expressing the pAg43BglC system were recovered by centrifugation (in a microfuge at 9,300 × *g* for 2 min at RT) and resuspended in 1 mL of 50 mM phosphate buffer (pH 7). Subsequently, 100 μL of the resuspended cells were mixed with 630 μL of the same buffer. Then, the cell suspension was allowed to equilibrate to the assay temperature for 5 min. The reaction was initiated via the addition of 100 mM pNPG (20 μL), and, after 5 min, the reaction was terminated by adding 2 M Na_2_CO_3_ (250 μL). The absorbance (at 400 nm) was measured after the cells were centrifuged in a microfuge at 9,300 × *g* for 2 min at RT, and the enzyme activity was calculated using the extinction coefficient for *p*-nitrophenol (18.5 mM^−1^) [21]. One pNPGase unit was defined as the amount of BglC attached to the cells such that the formation of pNP is catalyzed at a rate of 1 μmol per minute.

To measure the *β*-glucosidase activity of soluble BglC, 20 μL of the enzyme solution was mixed with 710 μL of 50 mM phosphate buffer (pH 7) and allowed to equilibrate to the assay temperature for 5 min. The reaction was initiated via the addition of 20 μL pNPG (100 mM), and, after 10 min, the reaction was terminated by adding 250 μL Na_2_CO_3_ (2 M). The absorbance (at 400 nm) was measured, and the enzyme activity was calculated using the extinction coefficient for *p*-nitrophenol (18.5 mM^−1^) [21]. One pNPGase unit was defined as the amount of BglC necessary to catalyze the formation of pNP at a rate of 1 μmol per minute. To measure the *β*-glucosidase activity present in the milieu of *E. coli* expressing the Ag43-BglC system, 4 mL of the supernatant was passed through a centrifugal filter unit (Amicon Ultra-4, Millipore Corporation, MA, USA) with a 10 kDa molecular weight cut-off. The *β*-glucosidase present in the supernatant was washed twice through the filter unit with 4 mL of 50 mM phosphate buffer (pH 7) to remove any unutilized cellobiose, which can interfere with the interaction of BglC with pNPG. Subsequently, the supernatant was concentrated in the filter unit to a volume of approximately 0.5 mL, and the activity was measured as already described.

### Analyses

Growth was analyzed spectrophotometrically as the optical density at 600 nm (DU-70, Beckman Instruments Inc., CA, USA) and converted to dry cell weight (DCW) per liter using a calibration curve (1 optical density = 0.37 g_DCW_ /L). The samples were centrifuged, and the cell-free culture broth was frozen until it was analyzed. The concentration of glucose was measured with an enzymatic analyzer (Model 2700, YSI Inc., OH, USA), whereas the cellobiose concentration was measured using the 3,5-dinitrosalicylic acid (DNS) method as described by Miller [33]. The ethanol was analyzed by gas chromatography using n-butanol as the internal standard (6850 Series GC System, Agilent, DE, USA). The concentrations of the cell biomass, ethanol and sugars were corrected for fermentation volume changes caused by the addition of base during culture.

Specific growth rates (μ) were calculated as the slopes of the linear regressions of the natural log of the cell mass versus time during the exponential growth phase. The ethanol yield (g ethanol/g carbon source) was estimated as a percentage of the theoretical maximum, taking into account that the theoretical yields obtained with glucose and cellobiose were 0.51 and 0.54, respectively. All cultures and enzyme assays were carried out at least in triplicate (the averages and standard deviations are shown in plots and tables).

## Competing interests

The authors declare that they have no competing interests.

## Authors’ contributions

IMG and AM participated in the design of this study. IMG constructed the plasmids and strains and performed the experimental work. CMA and BTM supported the development of cultures in mini-fermenters and the measurement of some enzymatic activities. IMG and AM analyzed the experimental data. IMG, GG and AM participated in the analysis of the results as well as in the writing and review of the manuscript. All authors have read and approved this manuscript.

## References

[B1] Caspeta L Buijs NAA Nielsen J The role of biofuels in the future energy supply Energy Environ Sci 2013 6 1077 1082 10.1039/c3ee24403b

[B2] Cardona CA Sánchez OJ Fuel ethanol production: process design trends and integration opportunities Bioresour Technol 2007 98 2415 2457 10.1016/j.biortech.2007.01.002 17336061

[B3] Olofsson K Bertilsson M Lidén G A short review on SSF - an interesting process option for ethanol production from lignocellulosic feedstocks Biotechnol Biofuels 2008 1 7 10.1186/1754-6834-1-7 18471273 PMC2397418

[B4] Castro E Faraco V Other Ethanologenic Microorganism Lignocellul Convers 2013 Springer-Verlag, Berlin, Heidelberg 152 168

[B5] Fernández-Sandoval MT Huerta-Beristain G Trujillo-Martinez B Bustos P González V Bolivar F Gosset G Martinez A Laboratory metabolic evolution improves acetate tolerance and growth on acetate of ethanologenic *Escherichia coli* under non-aerated conditions in glucose-mineral medium Appl Microbiol Biotechnol 2012 96 1291 1300 10.1007/s00253-012-4177-y 22669633

[B6] Wang X Yomano LP Lee JY York SW Zheng H Mullinnix MT Shanmugam KT Ingram LO Engineering furfural tolerance in *Escherichia coli* improves the fermentation of lignocellulosic sugars into renewable chemicals Proc Natl Acad Sci U S A 2013 110 4021 4026 10.1073/pnas.1217958110 23431191 PMC3593909

[B7] Edwards MC Henriksen ED Yomano LP Gardner BC Sharma LN Ingram LO Doran Peterson J Addition of genes for cellobiase and pectinolytic activity in *Escherichia coli* for fuel ethanol production from pectin-rich lignocellulosic biomass Appl Environ Microbiol 2011 77 5184 5191 10.1128/AEM.05700-11 21666025 PMC3147455

[B8] Merino ST Cherry J Progress and challenges in enzyme development for biomass utilization Adv Biochem Eng Biotechnol 2007 108 95 120 17594064 10.1007/10_2007_066

[B9] Moss-Acosta C Trujillo-Martinez B Orencio-Trejo M Martinez-Jimenez A Sequential thermochemical hydrolysis, enzymatic saccharification and fermentation to ethanol of stover from white corn with ethanologenic bacteria35th Symp Biotechnol Fuels Chem Soc Ind Microbiol 2013 EUA, Portland, Oregon

[B10] Muñoz-Gutiérrez I Martinez A Polysaccharide hydrolysis with engineered *Escherichia coli* for the production of biocommodities J Ind Microbiol Biotechnol 2013 40 401 410 10.1007/s10295-013-1245-y 23478881

[B11] Nicolay T Vanderleyden J Spaepen S Autotransporter-based cell surface display in Gram-negative bacteria Crit Rev Microbiol 2013 7828 1 15 10.3109/1040841X.2013.804032 23855358

[B12] Leyton DL Rossiter AE Henderson IR From self sufficiency to dependence: mechanisms and factors important for autotransporter biogenesis Nat Rev Microbiol 2012 10 213 225 10.1038/nrmicro2733 22337167

[B13] Muñoz-Gutiérrez I Oropeza R Gosset G Martinez A Cell surface display of a β-glucosidase employing the type V secretion system on ethanologenic *Escherichia coli* for the fermentation of cellobiose to ethanol J Ind Microbiol Biotechnol 2012 39 1141 1152 10.1007/s10295-012-1122-0 22638789

[B14] Wargacki AJ Leonard E Win MN Regitsky DD Santos CNS Kim PB Cooper SR Raisner RM Herman A Sivitz AB Lakshmanaswamy A Kashiyama Y Baker D Yoshikuni Y An engineered microbial platform for direct biofuel production from brown macroalgae Science 2012 335 308 313 10.1126/science.1214547 22267807

[B15] Gustavsson M Bäcklund E Larsson G Optimisation of surface expression using the AIDA autotransporter Microb Cell Fact 2011 10 72 10.1186/1475-2859-10-72 21917130 PMC3192670

[B16] Maurer J Jose J Meyer TF Autodisplay: one-component system for efficient surface display and release of soluble recombinant proteins from *Escherichia coli* J Bacteriol 1997 179 794 804 9006035 10.1128/jb.179.3.794-804.1997 PMC178762

[B17] Ramesh B Sendra VG Cirino PC Varadarajan N Single-cell characterization of autotransporter-mediated *Escherichia coli* surface display of disulfide bond-containing proteins J Biol Chem 2012 287 38580 38589 10.1074/jbc.M112.388199 23019324 PMC3493903

[B18] Kjaergaard K Hasman H Schembri MA Klemm P Antigen 43-mediated autotransporter display, a versatile bacterial cell surface presentation system J Bacteriol 2002 184 4197 4204 10.1128/JB.184.15.4197-4204.2002 12107137 PMC135209

[B19] Wells TJ Totsika M Schembri MA Autotransporters of *Escherichia coli*: a sequence-based characterization Microbiology 2010 156 2459 2469 10.1099/mic.0.039024-0 20447993

[B20] Van der Woude MW Henderson IR Regulation and function of Ag43 (flu) Annu Rev Microbiol 2008 62 153 169 10.1146/annurev.micro.62.081307.162938 18785838

[B21] Spiridonov NA Wilson DB Cloning and biochemical characterization of BglC, a β-glucosidase from the cellulolytic actinomycete *Thermobifida fusca* Curr Microbiol 2001 42 295 301 11178732 10.1007/s002840110220

[B22] Desai SH Rabinovitch-Deere CA Tashiro Y Atsumi S Isobutanol production from cellobiose in *Escherichia coli* Appl Microbiol Biotechnol 2014 98 3727 3736 10.1007/s00253-013-5504-7 24430208

[B23] Soma Y Inokuma K Tanaka T Ogino C Kondo A Okamoto M Hanai T Direct isopropanol production from cellobiose by engineered *Escherichia coli* using a synthetic pathway and a cell surface display system J Biosci Bioeng 2012 114 80 85 10.1016/j.jbiosc.2012.02.019 22561882

[B24] Tanaka T Kawabata H Ogino C Kondo A Creation of a cellooligosaccharide-assimilating *Escherichia coli* strain by displaying active beta-glucosidase on the cell surface via a novel anchor protein Appl Environ Microbiol 2011 77 6265 6270 10.1128/AEM.00459-11 21742905 PMC3165374

[B25] Henderson IR Owen P The major phase-variable outer membrane protein of *Escherichia coli* structurally resembles the immunoglobulin A1 protease class of exported protein and is regulated by a novel mechanism involving Dam and OxyR J Bacteriol 1999 181 2132 2141 10094691 10.1128/jb.181.7.2132-2141.1999 PMC93626

[B26] Seidle HF Marten I Shoseyov O Huber RE Physical and kinetic properties of the family 3 β-glucosidase from Aspergillus niger which is important for cellulose breakdown Protein J 2004 23 11 23 10.1023/B:JOPC.0000016254.58189.2a 15115178

[B27] Calsavara LP De Moraes FF Zanin GM Comparison of catalytic properties of free and immobilized cellobiase novozym 188 Appl Biochem Biotechnol 2001 91–93 615 626 10.1385/ABAB:91-93:1-9:615 11963890

[B28] Utrilla J Licona-Cassani C Marcellin E Gosset G Nielsen LK Martinez A Engineering and adaptive evolution of *Escherichia coli* for D-lactate fermentation reveals GatC as a xylose transporter Metab Eng 2012 14 469 476 10.1016/j.ymben.2012.07.007 22885034

[B29] Sambrook J Rusell D Molecular Cloning a Laboratory Manual 2001 Cold Spring Harbor Laboratory Press, Cold Spring Harbor, New York

[B30] Martinez A Grabar TB Shanmugam KT Yomano LP York SW Ingram LO Low salt medium for lactate and ethanol production by recombinant *Escherichia coli* B Biotechnol Lett 2007 29 397 404 10.1007/s10529-006-9252-y 17160622

[B31] Beall DS Ohta K Ingram LO Parametric studies of ethanol production form xylose and other sugars by recombinant *Escherichia coli* Biotechnol Bioeng 1991 38 296 303 10.1002/bit.260380311 18600763

[B32] Wood T Bhat K Methods for measuring cellulase activities Methods Enzymol 1988 160 87 112 10.1016/0076-6879(88)60109-1

[B33] Miller GL Use of dinitrosalicylic acid reagent for determination of reducing sugar Anal Chem 1959 31 426 428 10.1021/ac60147a030

